# Emerging medical therapies in crush syndrome – progress report from basic sciences and potential future avenues

**DOI:** 10.1080/0886022X.2020.1792928

**Published:** 2020-07-14

**Authors:** Ning Li, Xinyue Wang, Pengtao Wang, Haojun Fan, Shike Hou, Yanhua Gong

**Affiliations:** a Institute of Disaster Medicine, Tianjin University, Tianjin, China; b Tianjin Key Laboratory of Disaster Medicine Technology, Tianjin, China; c General Hospital of Tianjin Medical University, Tianjin, China

**Keywords:** Crush injury, crush syndrome, acute kidney injury, medical therapy, disaster medicine

## Abstract

Crush injury is a disease that is commonly found in victims of earthquakes, debris flows, mine disasters, explosions, terrorist attacks, local wars, and other accidents. The complications that arise due to the crush injury inflicted on victims give rise to crush syndrome (CS). If not treated in time, the mortality rate of CS is very high. The most important measure that can be taken to reduce mortality in such situations is to immediately start treatment. However, the traditional treatment methods such as fluid resuscitation, diuresis, and hemodialysis are not feasible enough to be carried out at the disaster scene. So there is a need for developing new treatments that are efficient and convenient. Because it is difficult to diagnose in the disaster area and reach the treatment equipment and treat on time. It has become a new research needs to be directed into identifying new medical treatment targets and methods using the etiology and pathophysiological mechanisms of CS. In recent years, a large number of new anti-oxidant and anti-inflammatory drug therapies have been shown to be highly efficacious in CS rat/mouse models. Some of them are expected to become specific drugs for the emergency treatment of a large number of patients who may develop CS in the aftermath of earthquakes, wars, and other disasters in the future. Hence, we have reviewed the latest research on the medical therapy of CS as a source for anyone wishing to pursue research in this direction.

## Introduction

Crush syndrome (CS) was first observed during the Messina earthquake in Italy in 1909 [1]. However, CS was not recognized as a clinical entity until a report by Bywaters and Beall during the World War II in 1941 [2] when victims from affected buildings appeared to be in good condition upon rescue, but died of kidney failure a few days later [3]. Since then, CS has been reported in a variety of disasters [4,5], and its clinical and pathological characteristics have become increasingly clear over the years.

CS is also known as traumatic rhabdomyolysis or Bywaters’ syndrome. It is mainly caused by direct or indirect trauma, which causes long-term crushing of muscle-rich parts such as limbs or torso, resulting in the compression and destruction of striated muscle cells. After release and restoration of the blood supply, the cell contents including nephrotoxicity myoglobin (Mb), urate, phosphate, potassium, etc. are released into the blood circulation [6], which eventually lead to myoglobinuria, acute kidney injury (AKI), electrolyte metabolic disorders, hypovolemic shock, multiple organ dysfunction syndrome (MODS), and so on [7].

Most medical therapies highlight the symptoms of circulatory shock, kidney failure, and arrhythmias in patients [8–10], and focus on early fluid resuscitation, forced diuresis and renal replacement therapy (RRT) which includes dialysis (hemodialysis or peritoneal dialysis), haemofiltration, haemodiafiltration and kidney transplantation [10–12]. For renal replacement therapy, continuous venovenous haemofiltration (CVVHF) is primarily used to remove myoglobin until the patient's kidney function returns or hemodialysis can be initiated [13]. However, most of the patients undergoing these medical therapies still suffer from systemic inflammatory response syndrome (SIRS) that frequently develops into multiple organ failure (MOF), eventually leading to death [14,15]. Therefore, CS is a life-threatening disease for which new medical therapies are promptly needed. Because it is difficult to diagnose in the disaster area and reach the treatment equipment and treat on time, there is an urgent need for safe and effective medical procedures to reduce the on-site mortality rate of CS [16], in addition to the aggressive fluid resuscitation in clinical practice. The current research on medical therapy for CS is particularly important. We need to constantly summarize valuable pre-clinical and emerging data to explore possible treatment approaches in the future. In this review, we have provided current research progress in the new medical therapies of CS in animal models (rat or mouse), to help explore future avenues.

## Chemical drugs

### Mitochondria-targeted anti-oxidants (SkQR1)

Koyner et al. [17] found that oxidative stress is critical for inducing acute kidney injury by rhabdomyolysis and ischemia/reperfusion, and that mitochondria are the source and target of excessive production of reactive oxygen species (ROS). Plotnikov et al. [18] found a mitochondria-targeted compound which is a combination of positively charged rhodamine and plastoquinone (SkQR1) that can reduce acute kidney injury in the rat model. The mitochondria-targeted anti-oxidant SkQR1 can induce some components of the renal protective pathway; it can increase the expression of erythropoietin and phosphorylate glycogen synthase kinase 3β in the kidney, which has direct anti-oxidant effects and can induce activation of the ischemic pre-processing signal pathway. Plotnikov et al. [19] also found that in addition to the direct anti-oxidant effects, SkQR1 also up-regulates protective signaling mechanisms. In other words, it not only protects the kidneys in the rat model of rhabdomyolysis, but also provides protection to the heart and brain. Therefore, the antioxidant treatment of CS patients is a very valuable research direction.

### Dexamethasone

The glucocorticoid dexamethasone (DXM) is a corticosteroid that can act as an anti-inflammatory, anti-toxic, anti-allergic and anti-rheumatic drug [20–23]. It is widely used in clinical practice and is easily absorbed through the digestive tract. Since the CS involves excessive inflammation in the body, researchers have tried to use DXM to treat CS rat models. Murata et al. [24–26] found that high-dose DXM injections exerted anti-inflammatory effects and reduced ischemia/reperfusion injury (IRI) through the PI3K-Akt-eNOS signaling pathway. This significantly improved the early survival rate of CS rat model. The authors also found that the intramuscular injection of DXM was not significantly different from intravenous treatment. However, in the actual application it is still necessary to evaluate the condition, transport, and treatment strategy of CS patients to determine the status of muscle injury and whether DXM is needed. The authors also showed that DXM therapy could prevent SIRS and the death caused by CS, and may help to develop new treatment strategies for CS. However, related cases reported that the use of steroids may cause rhabdomyolysis, which needs to be considered as a potential adverse effect and a limitation of dexamethasone [27–29].

### Allopurinol

Xanthine oxidase (XO) and xanthine dehydrogenase (XDH) are interconvertible forms of the same enzyme, xanthine oxidoreductase [30,31]. This is a key enzyme in the purine decomposition pathway that catalyzes the conversion of xanthine and hypoxanthine to uric acid (UA) [32,33]. During muscle contraction, the irreversible conversion of xanthine dehydrogenase (XDH) to its xanthine oxidase (XO) [34,35] plays an important role in producing ROS in ischemic conditions [36,37]. Allopurinol is an XO inhibitor and a UA-lowering agent commonly used in the treatment of gout [38,39]. Some animal studies have reported that glycerol-induced AKI can be effectively alleviated by inhibiting oxidative stress and apoptosis [40,41]. Studies by Gois et al. [42] showed that allopurinol treatment can reduce renal dysfunction by reducing oxidative stress (systemic, kidney and muscle), inhibiting apoptosis, reducing inflammatory cell infiltration, and increasing cell proliferation in rhabdomyolysis-related AKI rat model. If clinical research obtains positive results in the future, allopurinol treatment may become a new method for preventing and treating rhabdomyolysis-related AKI.

### Nitrite

Nitrite acts as a nitric oxide (NO) donor and nitrosating agent, which can be used as a signal molecule and gene regulator [43,44]. IRI is protected clinically by ischemic pre- and post-conditioning. Intravenous injection of nitrite inhibits IRI through related mechanisms mediated by NO [45,46]. Since the onset of CS is unpredictable, it is impractical to perform pharmacological pretreatment of CS-induced IRI with NO. However, it may be used as an on-site post-treatment agent immediately before reperfusion injury. With this in mind, Murata et al. [47–49] conducted research using a CS rat model. The results show that in addition to an expansion in conventional extracellular fluid volume and acid-base controlled infusion therapy, nitrite infusion is a promising drug intervention therapy that can treat CS by interfering with NO-mediated protective mechanisms. Therefore, the authors suggest that low-cost nitrite infusion therapy should be implemented clinically to prevent IRI and serve as a potential therapeutic agent for CS.

### Anisodamine

Anisodamine is a belladonna alkaloid, which is isolated from the traditional Chinese medicinal herb *Salvia miltiorrhiza* belonging to the Solanaceae family [50,51]. It is mainly used to relieve circulatory diseases in clinical conditions such as disseminated intravascular coagulation (DIC) and skeptic shock [52,53]. The research of Yu et al. [54] showed that anisodamine activated α 7 nicotinic acetylcholine receptor (α7nAChR) to increase the level of serum estradiol. This further enhanced insulin sensitivity in reducing serum potassium, which in turn helped to reduce the on-site mortality of the CS mouse model. These findings also encourage further research on anisodamine for the treatment of CS at the disaster site. Fan et al. [55] found that anisodamine treatment inhibited the increase of high mobility group box 1 protein (HMGB1) in CS rat and mouse models. However, the effect of anisodamine on proinflammatory cytokines in CS and its relationship with mortality needs further study [56].

### Astragaloside-IV

Astragaloside-IV is a biologically active astragaloside saponin isolated from astragalus [57,58]. It has been reported that astragaloside-IV has anti-inflammatory [59,60] and anti-oxidant [61,62] effects through anti-mitochondrial damage, and can prevent acute kidney [63] and tubular injury [64]. The therapeutic effect of astragaloside-IV on acute renal failure is consistent with the benefits of NO generation *via* endothelial nitric oxide synthase (eNOS) [65,66]. Therefore, the effect of astragaloside-IV is thought to be related to NO. In the early stage, the production of IRI is prevented through the protective effects of NO by inhibiting the systemic inflammatory response [48]. Murata et al. [67] showed that the direct and indirect anti-oxidant effect of astragaloside-IV on the kidney and the preventive effect on mitochondrial dysfunction and inflammatory response can significantly improve the survival rate of CS rat model by the rehydration treatment. This provides new insight into the prevention of CS-related renal failure by rehydration and resuscitation, or for the prognosis of CS by inhibiting the inflammatory response.

### Hydrogen sulfide (H_2_S)

Hydrogen sulfide (H_2_S) is a newly discovered gas signal molecule which is generated in the human body [68]. Low concentration of H_2_S plays a role in relaxing blood vessels, controlling blood pressure and maintaining homeostasis [69,70]. It also participates in regulating apoptosis, oxidative stress and inflammatory responses [71,72]. Teksen et al. [73] found that applying exogenous H_2_S (NaHS) to treat the CS rat model could effectively reduce the expression of kidney injury molecule-1 (KIM-1), neutrophil gelatinase-related lipoprotein (NGAL), TNF-α, TGF-β, and total oxidant, therefore improving kidney anti-oxidant levels. Meanwhile, the levels of blood urea nitrogen (BUN), creatinine (Cre), and creatine kinase (CK) also decreased with the application of NaHS. The results of this study indicated that the application of exogenous H_2_S (NaHS) can effectively alleviate acute renal failure in CS rat model through anti-inflammatory, anti-oxidant, and anti-apoptotic effects. This study lays the foundation for the future application of H_2_S in the clinical treatment of CS. However, further pathological and pharmacological studies are needed to confirm the safety, safe dosage range, and the efficacy of CS of using H_2_S.

### Bardoxolone methyl (BM)

Bardoxolone methyl (BM) is a semi-synthetic oleanane triterpenoid derivative. Clinical studies have shown that BM can achieve anti-inflammatory and anti-oxidant effects by activating nuclear factor erythroid 2-related factor 2 (Nrf2) and inhibiting nuclear factor-κB (NF-κB) [74] by effectively improving glomerular filtration rate in patients with chronic kidney disease (CKD) [75]. Kadioglu et al. [76] showed that BM treatment can improve AKI in the CS rat model. These results also suggested that BM may reduce crush kidney injury by reducing the expression of TNF-α and TGF-β. In addition, the role of BM is not limited to the kidney. It has systemic anti-oxidant, anti-inflammatory, and anti-apoptotic effects and can also reduce other complications related to CS. However, given that Vaziri et al. [77] reported that BM analogues have a dose-dependent toxic effect on the progression of CKD, the use of BM in large doses may cause serious side effects. In the future, further experiments are needed to explore the safety of BM treatment in CS patients.

### N-(2-hydroxyphenyl) acetamide

N- (2-hydroxyphenyl) acetamide (NA-2), also known as 2-acetaminophenol, is a synthetic compound and a derivative of salicylic acid [78]. It is an effective anti-inflammatory drug in rat arthritis model *in vivo* [79,80]. Siddiqui et al. [81] found that NA-2 and its gold nanoparticle conjugation (NA-2-AuNPs) attenuates the inflammation and kidney injury in glycerol-induced AKI mouse model through anti-oxidant and anti-inflammatory mechanisms. NA-2 prevents kidney injury caused by rhabdomyolysis by reducing BUN and Cre levels. Compared with NA-2 alone, NA-2-AuNPs protect renal structure at low doses. This study also found that both NA-2 and NA-2-AuNPs can retain the renal tubule brush border and actin cytoskeleton. The kidney protection mechanisms of these compounds seem to downregulate the expression of cyclooxygenase-2 (COX-2), NF-κB and iNOS, and upregulate the expression of HO-1 and KIM-1, thereby protecting the kidney from inflammation and oxidants.

### Ulinastatin

Ulinastatin is an acidic glycoprotein purified from the urine and blood of healthy people [82]. It is a multifunctional serine protease inhibitor that inhibits a variety of serine proteases, such as trypsin, thrombin, chymotrypsin, kallikrein and plasmin [83,84]. Yang et al. [85] found that the early administration of ulinastatin to CS rat model can reduce CS-induced AKI and reduce inflammation. Ulinastatin can also significantly reduce the levels of BUN, CK, Cre, Mb, and K^+^ in the serum of CS rat model. It also inhibits the infiltration of inflammatory cells, reduces sarcomere rupture in compressed muscle tissues, reduces glomerular hyperemia and edema, and reduces the amount of Mb in kidney tissue. At the same time, the ratio of regulatory T (Treg) cells in CD4^+^ T cells after treatment with ulinastatin was significantly higher than that of the crush injury group, while the expression of IL-17 decreased. Therefore, the authors believe that Ulinastatin may play a renal protective role by regulating the balance between Th17 and Treg cells.

## Biological agents

### Recombinant human erythropoietin (rhEPO)

Erythropoietin (EPO) is a pleiotropic cytokine and a glycoprotein of about 34 kDa. It was originally thought to play a role in erythropoiesis [86,87] and has been used in the treatment of chronic kidney disease with anemia and cancer chemotherapy for 20 yrs [88]. Recently, studies have found that EPO and its receptor (EPO-R) interact in a variety of non-hematopoietic tissues to induce cytoprotective responses [89,90]. Yang et al. [91] found that recombinant human erythropoietin (rhEPO) could inhibit the activity of NF-κB and iNOS, reduce the expression of BUN, Cre, GOT, GPT, and CPK which are kidney injury markers after rhabdomyolysis, and relieve AKI induced by rhabdomyolysis in rats. Wang et al. [92] showed that EPO could alleviate kidney injury by reducing the recruitment of macrophages *in vivo* and promoting the transformation of M2 macrophage phenotypes. They also confirmed that EPO could directly inhibit the pro-inflammatory response of M1 macrophages *in vitro* and promote the expression of M2 markers. These findings may optimize the treatment of sterile kidney injury with EPO. Zhou et al. [93] further proved that rhEPO has immunomodulatory abilities, and confirmed that it plays a therapeutic role in CS by regulating the TLR4/NF-κB signaling pathway in macrophages.

### Macrophage surface molecule mac-1 inhibitor: Lactoferrin (Lf)

In recent years, many studies have confirmed that macrophages are involved in the pathogenesis of rhabdomyolysis syndrome, but the exact molecular mechanism is still unclear [94–96]. Okubo et al. [97] found that macrophages released extracellular traps (ETs) containing DNA fibers and granular proteins in a mouse model of rhabdomyolysis syndrome. Heme-activated platelets released by necrotic muscle cells during rhabdomyolysis syndrome interact with Mac-1 on macrophages, promoting the production of macrophage extracellular traps (METs) by increasing intracellular ROS production and histone citrullination. The authors speculated that these METs may cause kidney damage through the binding of Toll-like receptors to kidney cells. The results of this study suggested that the use of Mac-1 inhibitor (Lactoferrin) or direct inhibitor of METs can prevent platelet-mediated MET formation and related renal tubular injury in rhabdomyolysis-induced AKI. This method may develop into a new treatment strategy for CS.

### Antibody treatment: anti-HMGB1 antibody

High mobility group box 1 protein (HMGB1) is a ubiquitous non-histone chromosomal protein that can be passively released by necrotic cells and be actively secreted by inflammatory cells [98,99]. It is considered to be an important extracellular medium for systemic inflammation and plays a key role in the pathogenesis of systemic acute inflammation [100,101]. Shimazaki et al. [15] showed that HMGB1 is released immediately after crush injury and plays a pro-inflammatory effect. Administration of the anti-HMGB1 antibody can reduce the inflammatory response by blocking the action of extracellular HMGB1, thereby reducing organ damage and improving survival rate [15]. Therefore, HMGB1 seems to be a new therapeutic target. In addition, previous studies have shown that HMGB1 can indirectly increase the production of TNF-α [102] and upregulate the level of c-Jun N-terminal kinase (JNK) which is a stress-activated protein kinase [103]. Zhang et al. [104] also found that the administration of anti-HMGB1 antibody, anti-TNF-α antibody and JNK inhibitor SP600125 can reduce renal cortical cell apoptosis. This suggests that JNK and TNF-α may collectively participate in the positive feedback cycle of CS, leading to the increase of renal cortical cell apoptosis and further kidney damage. HMGB1 in the muscle may also act as a potential trigger.

### Antibody treatment: anti-RAGE antibody

Receptor for advanced glycation end products (RAGE) is a newly discovered pattern recognition receptor *in vivo* that regulates inflammation [105]. Studies have found that RAGE is widely involved in the pathological processes of many diseases and the pathogenesis of acute inflammatory diseases, and is closely related to Alzheimer’s disease, pneumonia, tumors and diabetes [105]. It is reported that HMGB1 can promote the signaling process of RAGE. Therefore, it is reasonable to believe that RAGE is closely related to the body's inflammatory response after crush injury [15]. Matsumoto et al. [106] investigated the role of anti-RAGE antibodies in the CS rat model. They found that intravenous injection of anti-RAGE antibodies in rats, before releasing compression, can reduce the degree of inflammatory response, prevent the development of MOF and facilitate the prognosis of CS.

### Cell therapy: mesenchymal stem cells treatment

Mesenchymal stem cells (MSCs) are pluripotent stem cells that have the ability to self-renew and differentiate into lineages of mesenchymal cells [107,108]. MSCs can be isolated from various tissues and are easy to culture *in vitro* [109]. Almeida et al. [110] showed that MSCs could protect the kidney injury induced by rhabdomyolysis in mice. Although some researchers have observed that migration and trans-differentiation into functional parenchymal cells of MSCs play a repairing role, its beneficial effects are mainly due to its paracrine properties. Duffy et al. [111] believe that the presence of MSCs can promote the accumulation of protective M2 macrophages in the kidney, increase the production of anti-inflammatory IL-10, and reduce the expression of IL-6 and TNF-α. Geng et al. [112] showed that MSC can improve rhabdomyolysis-induced AKI by activating macrophages to the M2 phenotype, thereby promoting the transition of renal tubules from injury to repair and accelerating the recovery period of rhabdomyolysis mouse model. This finding provides new clues in exploring the beneficial mechanisms of MSCs on AKI. These characteristics of MSCs can provide effective and innovative therapies for the treatment of acute and chronic kidney disease. However, more research is needed on MSCs-related therapies to ensure their clinical application for CS patients in the future.

### Cell therapy: carbon monoxide-enriched red blood cell (CO-RBC)

To improve the expression level and activity of cytochrome P450 during major bleeding, Ogaki et al. [113] developed a carbon monoxide (CO) delivery system. CO is bound to hemoglobin molecules in RBCs by putting CO gas through the preparation of RBC to create CO-enriched red blood cells (CO-RBC). It may become a novel form of cell therapy. Subsequently, Taguchi et al. [114] also indicated that CO-RBC has potential as a practical therapeutic agent for CS-related catastrophic kidney disease. The CO-RBC therapy can reduce the oxidation of myoglobin in the kidneys and the degradation of cytochrome P450, which can inhibit the production of free heme and hemoglobin and have protective effects for the kidneys. At the same time, researchers have discovered that CO-RBC can also function as a primitive O_2_-RBC after releasing CO. Most patients suffering from compression by heavy objects also have traumatic hemorrhage caused by falling stones and other reasons. Therefore, CO-RBC treatment is expected to be a very practical therapy in major disasters against CS and massive hemorrhage.

## Other treatment

### Icing treatment-liquid infusion therapy combined with icing

In recent years, there has been a controversy over the potential benefits of muscle healing after various trauma-induced damages. However, research has shown that lower tissue temperature is the most powerful intervention for limiting local IRI, which may prevent mitochondrial damage and reduce the severity of IRI. Murata et al. [115] indicate that icing treatment can inhibit the effect of vasoconstriction on potassium concentrations, have anti-inflammatory effects in the affected cardiomyocytes, and improve mitochondrial function. This study demonstrated that icing treatment after crush injury in rats can extend survival by inhibiting the increase of blood potassium concentration in the emergency. However, because CS involves multiple symptoms and rapid deterioration at the same time, this treatment cannot significantly improve the overall results. The icing treatment could suppress the acute inflammation reaction effectively, which can be improved by combining it with other fluid infusion therapies.

## Conclusions and perspectives

In recent years, various disasters and accidents have been occurring frequently. The CS caused by disaster ruins has caused widespread concern. With the occurrence and high mortality rate of patients with CS, traditional treatments have not yet met the clinical needs. It is necessary to develop new treatments that are efficient and convenient. Due to the pathophysiological mechanisms of CS, the efficacy of new anti-oxidant and anti-inflammatory treatments have been shown in the animal model of CS. Although the potential CS medical treatments in clinical application are few and need to be studied further to ensure their security in the future clinical application, the animal research on drug treatments of CS to the early efficient treatment at the scene for patients with CS in the future is of considerable significance. We have summarized the research on medical therapy for CS (Table 1). More importantly, after the in-depth study of the above medical therapy, it is expected to be used in emergency situations, before or during decompression of injured patients at the disaster site. This can make up for the inadequacy of professional medical equipment (such as CVVHF) on the scene.

**Table 1.** Detail information of different drug for crush syndrome therapy.

Drug category	Drug/strategy	Protective mechanism	Specie	Author	Author Country	Journal Year	References
Chemical drugs	Mitochondria-targeted anti-oxidants (SkQR1)	Anti-oxidant, activate ischemia pre-processing signal pathway	- Rat	Koyner et al. Plotnikov et al.	USA Russia	2008 2011	[17,18]
	Dexamethasone	Anti-inflammatory, reduce IRI through the PI3K-Akt-eNOS signal pathway	Rat Rat Rat	Murata et al.	Japan	2016 2015 2013	[24–26]
	Allopurinol	Reduce oxidative stress, inhibit apoptosis, reduce inflammatory cell infiltration, increase cell proliferation	Rat	Gois et al.	Brazil	2016	[42]
	Nitrite	Inhibit IRI relate to NO	Rat Rat -	Murata et al.	Japan	2012 2017 2018	[47–49]
	Anisodamine	Activate α7nAChR, reduce serum potassium, inhibit HMGB1	Mouse Rat	Yu et al. Fan et al.	China China	2019 2016	[54,55]
	Astragaloside-IV	Anti-oxidant, prevent mitochondrial dysfunction and inflammation response	Rat	Murata et al.	Japan	2017	[67]
	Hydrogen sulfide (H_2_S)	Anti-inflammatory, anti-oxidant, and anti-apoptotic	Rat	Teksen et al.	Turkey	2019	[73]
	Bardoxolone methyl (BM)	Anti-inflammatory, anti-oxidant, anti-apoptotic	Rat	Kadioglu et al.	Turkey	2019	[76]
	N-(2-hydroxyphenyl) acetamide	Downregulate (COX-2, NF-κB , iNOS), upregulate (HO-1, KIM-1), aiti-inflammatory, anti-oxidant, retain the renal tubule brush border and actin cytoskeleton	Mouse	Siddiqui et al.	Pakistan	2019	[81]
	Ulinastatin	Reduce inflammation, regulate the balance between Th17 and Treg cells	Rat	Yang et al.	China	2020	[85]
Biological agents	Recombinant human erythropoietin (rhEPO)	Inhibit NF-κB and iNOS, regulate TLR4/NF-κB to promote M2 macrophages phenotypes	Rat Mouse Mouse	Yang et al. Wang et al. Zhou et al.	Taiwan China China	2012 2017 2020	[91,92,93]
	Macrophage surface molecule Mac-1 inhibitor: Lactoferrin (Lf)	Prevent platelet-mediated MET formation and related renal tubular injury	Mouse	Okubo et al.	Japan	2018	[97]
Antibody treatment	Anti-HMGB1 antibody	Blocking HMGB1, reduce inflammatory response and renal cortical cell apoptosis	Rat Mouse	Shimazaki et al. Zhang et al.	Japan China	2012 2017	[15,104]
	Anti-RAGE Antibody	Reduce inflammatory response, prevent the development of MOF	Rat	Matsumoto et al.	Japan	2017	[106]
Cell therapy	Mesenchymal stem cells (MSC) treatment	Promote M2 macrophages phenotype	- Mouse	Almeida et al. Duffy et al. Geng et al.	Brazil Ireland	2013 2014 2014	[110,111,112]
	Carbon monoxide-enriched red blood cell (CO-RBC)	Reduce the oxidation, degrade cytochrome P450, inhibit free heme and hemoglobin	Rat	Taguchi et al.	Japan	2020	[114]
Other treatment	Icing treatment	Inhibiting the increase of blood potassium concentrations and suppress acute inflammation reaction	Rat	Murata et al.	Japan	2020	[115]

Abbreviation: SkQR1: Mitochondria-targeted anti-oxidants; IRI: ischemia/reperfusion injury; H_2_S: hydrogen sulfide; BM: Bardoxolone methyl; COX-2: Cyclooxygenase-2; NF-κB: Nuclear factor kappa-B; iNOS: Inducible nitric oxide synthase; HO-1: Heme oxygenase-1; KIM-1: Kidney injury molecule-1; rhEPO: Recombinant human erythropoietin; HMGB1: High mobility group box 1 protein; RAGE: Receptor for advanced glycation endproducts; MOF: multiple organ failure; MSC: Mesenchymal stem cells; CO-RBC: Carbon monoxide-enriched red blood cell.

In the current investigation of the pathogenesis of CS and potential therapeutic targets at the cellular, organelle and molecular level have become a new research direction. The recent trend is that the medical treatment of CS is gradually changing from the current supportive and symptomatic treatment to prevention and etiological treatment. We recommend adopting individualized and precise medical treatment methods.
